# Mechanochemical endovenous ablation versus radiofrequency ablation in the treatment of primary small saphenous vein insufficiency (MESSI trial): study protocol for a randomized controlled trial

**DOI:** 10.1186/1745-6215-15-421

**Published:** 2014-10-29

**Authors:** Doeke Boersma, Ramon RJP van Eekeren, Hans JC Kelder, Debora AB Werson, Suzanne Holewijn, Michiel A Schreve, Michel MPJ Reijnen, Jean Paul PM de Vries

**Affiliations:** Department of Vascular Surgery, St Antonius Hospital, Koekoeklaan 1, 3435 CM Nieuwegein, The Netherlands; Department of Surgery, Rijnstate Hospital, Wagnerlaan 55, 6815 AD Arnhem, The Netherlands; Department of Methodology and Statistics, St Antonius Hospital, Koekoeklaan 1, 3435 CM Nieuwegein, The Netherlands; Department of Surgery, Rode Kruis Hospital, Vondellaan 13, 1942 LE Beverwijk, The Netherlands

**Keywords:** Mechanochemical ablation, ClariVein, radiofrequency ablation, varicose vein, therapy, treatment, MOCA, outcome

## Abstract

**Background:**

Minimally invasive endothermal techniques, for example, radiofrequency ablation (RFA), have revolutionized the treatment of insufficient truncal veins and are associated with an excellent outcome. The use of thermal energy requires the instillation of tumescent anesthesia around the vein. Mechanochemical endovenous ablation (MOCA™) combines mechanical endothelial damage, using a rotating wire, with simultaneous infusion of a liquid sclerosans. Tumescent anesthesia is not required as no heat is used. Prospective studies using MOCA™ in both great and small saphenous veins showed good anatomical and clinical results with fast postoperative recovery.

**Methods/Design:**

The MESSI trial (Mechanochemical Endovenous ablation versus radiofrequency ablation in the treatment of primary Small Saphenous vein Insufficiency) is a multicenter randomized controlled trial in which a total of 160 patients will be randomized (1:1) to MOCA™ or RFA. Consecutive patients with primary small saphenous vein incompetence, who meet the eligibility criteria, will be invited to participate in this trial. The primary endpoint is anatomic success, defined as occlusion of the treated veins objectified with duplex ultrasonography at 1 year follow-up. Secondary endpoints are post-procedural pain, initial technical success, clinical success, complications and the duration of the procedure. Initial technical success is defined as the ability to position the device adequately, treat the veins as planned and occlude the treated vein directly after the procedure has been proven by duplex ultrasonography. Clinical success is defined as an objective improvement of clinical outcome after treatment, measured with the Venous Clinical Severity Score (VCSS). Power analyses are conducted for anatomical success and post-procedural pain.

Both groups will be evaluated on an intention-to-treat principle.

**Discussion:**

The hypothesis of the MESSI trial is that the anatomic success rate of MOCA™ is not inferior to RFA. The second hypothesis is that post-procedural pain is significantly less after MOCA compared to RFA.

**Trial registration:**

Trial registration: NTR4613 Date of trial registration: 28 May 2014.

## Background

Varicose veins are common and symptoms range from cosmetic complaints to venous ulcers. The incidence increases with age and women are more affected than men. The majority of venous associated complaints are due to great saphenous vein (GSV) insufficiency; however, in 15% of patients, small saphenous vein (SSV) insufficiency is the main cause [1–3].

During the last decade, minimally invasive endothermal catheter modalities took over the role of surgical therapy in the treatment of small saphenous insufficiency. Surgery has been associated with high recurrence rates (25 to 60%), nerve injuries, and significant postoperative complications [4–10]. Success rates after endothermal catheter techniques, for example, endovenous laser ablation (EVLA) and radiofrequency ablation (RFA), are excellent. At 1 year follow-up, the anatomical success rate of RFA is 88% [10]. RFA is associated with fewer procedure-related symptoms, superior cosmetic results and earlier resumptions of daily activity compared to traditional surgical procedures [11, 12]. However, to perform endothermal techniques, tumescent anesthesia is essential. Furthermore, heat-induced nerve injury and post-procedural pain is inherent to endothermal ablation and can become chronic. Nerve injuries after endothermal ablation of SSV are seen in up to 11% [13].

Mechanochemical endovenous ablation (MOCA™), using the ClariVein™ system (Vascular Insights LLC, Madison, CT, USA) was introduced in Europe in 2010. Mechanical injury to the endothelium by a rotating wire at the tip of a catheter is combined with an infusion of liquid sclerosans, without the use of tumescent anesthesia. In Europe, the ClariVein™ device was registered on April 26, 2010, CE 558723.

Recently, the authors published the first results of MOCA™ in SSV. Occlusion rates in 50 patients were 100% at 6 weeks follow-up and up to 97% at 1 year. In this cohort of SSVs, no major complications, including nerve injury, were seen. Minor complications, such as localized ecchymoses and superficial thrombophlebitis were seen in 12 to 14%. The Venous Clinical Severity Score (VCSS), an objective measure for varicose vein-specific symptoms, improved significantly from 3 (IQR 2 to 5) before treatment to 1 (IQR 1 to 3, *P* <0.001) at 6 weeks follow-up. Clinical results were retained at 1 year after treatment (VCSS 1, IQR 1 to 2, *P* <0.001). Patients were satisfied after treatment [14]. Several other studies reported promising data on feasibility, safety, anatomical and clinical success regarding MOCA treatment in GSV insufficiency, without major complications [15–17].

## Method/Design

### Study design

The multicenter randomized clinical MESSI trial is designed to compare MOCA™ and RFA in the treatment of SSV insufficiency. Patients with primary SSV insufficiency, meeting eligibility criteria, will be included at the outpatient clinics of the participating hospitals after giving written informed consent. The procedures are performed or supervised by dedicated vascular surgeons, who have performed over 20 procedures of both treatment modalities.

The following Dutch vascular centers are participating in the MESSI trial: St. Antonius Hospital Nieuwegein, Rijnstate Hospital Arnhem and Rode Kruis Hospital Beverwijk.

### Study objectives

The aim of the study is to show that MOCA™ has no inferior anatomical success compared to RFA at 1 year follow-up. The secondary aim is to evaluate post-procedural pain, which is hypothesized to be significantly less after MOCA™.

### Sample size calculation

Power analysis is calculated for anatomical success at 1 year follow-up after endovenous treatment of the SSV. The calculation is based on the hypothesis that MOCA™ will have no inferior occlusion rates at 1 year after treatment compared to RFA. The meta-analysis by Van de Bos *et al*. showed anatomical success rates of 88% after RFA [10]. Our recent study on MOCA™ in SSV showed an occlusion rate of 97% in the group treated according to our current study protocol [17]. A Chi-square test with a one-sided 0.05 significance has a power of 80% to observe no difference between both groups, when each group consists of 74 patients (non-inferiority principle, range 2%). The study population of both groups will consist of 148 patients. Corrected for 7.5% lost to follow-up, the total study population will consist of 160 patients.

Pain is an important secondary outcome parameter. A sample size calculation is also performed for this endpoint based on the hypothesis that MOCA™ will have lower post-procedural pain scores, as measured by a 100-point pain score, during the first two weeks after surgery. To evaluate a 30 percent reduction in post-procedural pain, 58 patients per group are needed (alpha 5%, power 80%). This analysis will be performed after inclusion and randomization of at least 58 patients in each group.

### Primary endpoints

The primary endpoint is anatomic success after treatment of SSV insufficiency with MOCA™ or RFA at 1 year follow-up. Anatomic success is defined as occlusion of the treated vein and proven with duplex ultrasonography.

### Secondary endpoints

Post-procedural pain is evaluated using a 100-millimeter visual analog scale (VAS) during the first 2 weeks after treatment. The other secondary endpoints are initial technical success, clinical success, pain during treatment, complications and duration of the procedure. Initial technical success is defined as the ability to position the device adequately, treat the veins as planned and occlude the treated vein directly after the procedure proven by duplex ultrasonography. Clinical success is measured using the Venous Clinical Severity Score (VCSS). Complications related to the endovenous treatment, that occur within 30 days after treatment, are divided between major complications (deep venous thrombosis, pulmonary embolism, nerve injury, skin burn), minor complications (ecchymosis, superficial thrombophlebitis, hyperpigmentation, wound infection, prolonged pain >1 week) and sclerosans-related complications. Additionally, all endpoints will be evaluated at 2 and 5 years post-treatment.

### Ethical considerations

A patient, who meets the inclusion criteria, will be fully informed about the trial and provided with a patient information and informed consent form. Patients willing to participate in the study are included after signing the informed consent form. This study is conducted in accordance with the principles of the Declaration of Helsinki and Good Clinical Practice guidelines. The study protocol has been approved by the medical ethical committee in Nieuwegein, (VCMO NL42781.100.13) and the local institutional board of each participating center (LHC Rijnstate Arnhem/Commissie Lokale Uitvoerbaarheid RKZ Beverwijk).

### Safety and quality control

#### Data safety monitoring board

The Data Safety Monitoring Board (DSMB) is composed of three independent physicians: two vascular surgeons and one dermatologist. The DSMB will review safety and provide recommendations regarding the conduct of the study to the steering committee and to the accredited medical ethical committee (VCMO Nieuwegein) that approved the study protocol. An interim safety analysis will be performed after treatment and at the 4-week follow-up of the first 80 patients.

#### Adverse and severe adverse events

Adverse events (AE) are defined as any undesirable experience occurring to a participant during the study, whether or not considered related to the investigational device. This definition includes events occurring during hospital stay and up to 30 days of follow-up. Underlying disease that was present at the time of enrollment is not reported as an AE, but any increase in the severity of the underlying disease will be reported as an AE. All AEs will be monitored from the time of enrollment through the 30-day follow-up visit. Adverse events will be recorded on the case record forms. A description of the event, including the start date, end date, action taken, and the outcome will be provided.

A severe adverse event is any event leading to death, deep venous thrombosis, and neurological complications.

Data on AEs will be reported to the DSMB and to the accredited medical ethical committee via ‘Toetsingonline’ on the website of the Central Committee on Research involving Human Subjects (CCMO, http://www.ccmo.nl).

### Inclusion criteria

Inclusion criteria are: unilateral primary SSV insufficiency; C2 to C5 varicose veins; diameter of the SSV at the saphenopopliteal junction ≥3 or ≤12 millimeter; age between 18 and 80 years and written informed consent.

### Exclusion criteria

Exclusion criteria are: C6 varicose veins; previous surgery or endovenous treatment for insufficient SSV of the ipsilateral leg; oral anticoagulants; pregnancy or lactation; previous deep venous thrombosis; immobilization; contraindication or known allergy for sclerosans; coagulation disorders or an increased risk of thromboembolism; severe renal insufficiency (eGFR <30 ml/min) and/or severe liver insufficiency (leading to coagulation disorders).

### Recruitment

**Figure 1:**
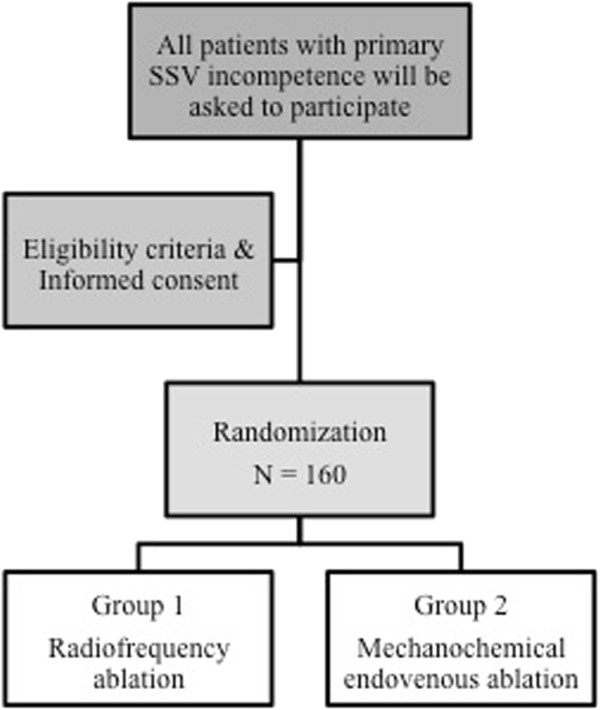
**Flow chart.** Legend: This figure illustrates the study design. A total of 160 patients will be randomized to radiofrequency ablation or mechanochemical endovenous ablation.

A total of 160 patients with primary SSV insufficiency will be included in the MESSI trial after signing informed consent (Figure 1). Before treatment begins, a vascular surgeon or dedicated physician assistant will perform a physical examination. The Clinical Etiology Anatomy Pathophysiology (CEAP) score [18] and VCSS [19] are determined. Insufficiency of the SSV is defined by duplex ultrasound as a retrograde flow >0.5 seconds after calf compression while standing [20].

After randomization, the SSV is obliterated in an outpatient setting according to the following: study arm 1: radiofrequency ablation (RFA) or study arm 2: mechanochemical endovenous ablation (MOCA™).

### Randomization

Patients will be randomized directly after inclusion by the treating vascular surgeon or physician assistant to one of the treatment arms, using a validated web-based randomization tool (Research Manager, NOVA Business Software, Zwolle, The Netherlands). The 1:1 randomization is performed by blocks of ten with stratification for participating centers.

### Treatment details

#### Radiofrequency ablation (RFA)

During RFA, radiofrequency energy is used to heat the vein wall of the SSV. The catheter is inserted into the vein and direct energy is delivered to the endothelium with collapsing and sealing of the vein as effect. During the MESSI study, all participating centers are using the VNUS™ ClosureFAST™ catheter (VNUS Medical Technologies, San Jose, California, USA). The VNUS™ ClosureFAST™ catheter contains a 7-cm long heating element at the end of the catheter, used for segmental ablation. Every 7-cm segment is treated during a 20-second treatment cycle in which a temperature of 120 degrees is maintained. Only the most proximal segment is treated during the two cycles [21].

The insufficient SSV is punctured distally under ultrasound-guidance and a guide wire is inserted. An introducer sheath is placed over the guide wire. Subsequently, the RFA catheter is introduced and positioned approximately 2 cm distal to the saphenopopliteal junction using ultrasound guidance. Then, tumescent anesthesia is delivered along the entire SSV to be treated. The target vein is compressed circumferentially and positioned at least 1 cm below the skin, due to the tumescence.

#### Mechanochemical endovenous ablation

**Figure 2:**
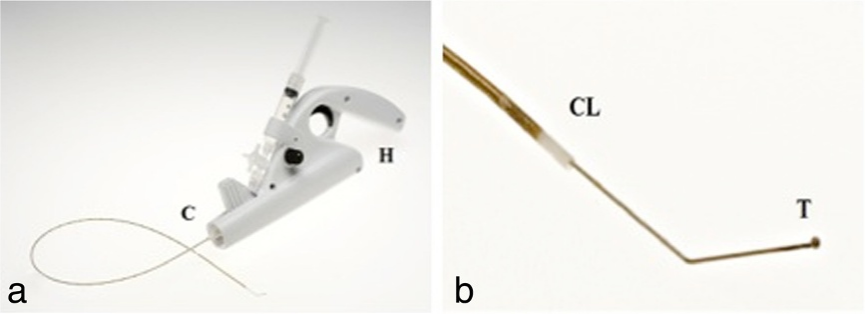
**ClariVein™ device.** Legend: **(a)** The ClariVein™ device consists of motor unit (H) and infusion catheter (C). **(b)** The dispersion tip (T) protrudes with angulated tip from catheter (CL). Acknowledgement: Figure 1 is reproduced with permission from the Journal of Endovascular Therapy. Copyright 2011. International Society of Endovascular Specialists.

The MOCA™ technique has been previously described [15]. Briefly, the ClariVein™ device is a disposable 2.6 F single-lumen catheter for infusing liquid sclerosans. A metal wire, fitted distally with a small ball, runs through the catheter (Figure 2). The motorized handle unit rotates this wire at 3,000 rpm. The purpose of this wire is to create intimal injury, to induce vasospasm and disperse the liquid sclerosans.

The ClariVein™ catheter is introduced into the distal SSV through a 4 F micropuncture sheath. The tip of the dispersion wire is positioned 1 cm distal to the saphenopopliteal junction (SPJ). To induce spasm of the proximal SSV, the rotating wire is activated for 10 seconds. Then, the activated catheter with rotating tip is steadily withdrawn at the rate of 1 cm every 7 seconds, simultaneously dispersing liquid polidocanol (Aethoxysklerol™, KreusslerPharma, Wiesbaden, Germany). In the proximal 10 cm of the SSV 2 mL 3% polidocanol is used, the remaindering insufficient SSV is treated with 1.5% polidocanol. The total amount of used liquid sclerosans will be documented and will not exceed the allowed daily dose of 2 mg/kg/day.

#### Both treatments

After treatment, both the deep venous system and the treated SSV are scanned by ultrasound.

A compression stocking (20 to 30 mmHg) will be applied for 24 hours continuously and for 2 weeks during the daytime. Patients are asked to resume normal activities immediately after the procedure. No concomitant phlebectomy or sclerotherapy are performed during the first 4 weeks of follow-up. Concomitant treatment after 4 weeks is reported in trial results.

### Follow-up

After 4 weeks, 1 year, 2 years and 5 years, patients are seen at the outpatient clinic to determine the anatomical and clinical success (Table 1). Ultrasound duplex imaging, VCSS, CEAP score and health status is measured at all aforementioned time points. Ultrasound duplex imaging is done according to a standardized protocol for all participating hospitals.

**Table 1:** **Follow-up chart**

	Study period					
	Screening	Procedure	4 wk	1 yr	2 yr	5 yr
**Outpatient visit**	X		X	X	X	X
**Physical examination**	X		X	X	X	X
**Informed consent**	X					
**Inclusion criteria**	X					
**Randomization**	X					
**CEAP/VCSS**	X		X	X	X	X
**Ultrasound**	X		X	X	X	X
**Pain score**		X	X			
**AVVQ**	X		X	X	X	X
**SF-36**	X		X	X	X	X

AVVQ, Aberdeen Varicose Vein Questionnaire; CEAP, Clinical Etiology Anatomy Pathophysiology classification; SF-36, Short Form 36-Item Health Survey; VCSS, Venous Clinical Severity Score.

Post-procedural pain is evaluated using a linear VAS score of 0 to 100 mm during the 2 weeks after treatment. After 4 weeks follow-up, any small branch varicosities may be treated when indicated.

### Health status measurement

Health status will be measured according to the following:

1. Short Form-36 (SF-36) is a multidimensional measurement of general health. It yields eight domains of functional health and well-being scores.
2. Aberdeen Varicose Vein Questionnaire [22] (AVVQ) is a validated disease-specific health status measurement for chronic venous insufficiency (patient reported outcome).

Both the SF-36 and AVVQ questionnaires are completed pre-procedural and after 4 weeks, 1 year, 2 years and 5 years of follow-up.

### Data collection and management

All data will be collected at each participating treating center by case report forms (CRFs). Photocopies of the CRFs will be sent to the coordinating investigator (DB). The data will be entered in a validated data management system (Research Manager, NOVA Business Software, Zwolle, The Netherlands) and controlled by an independent monitor. The participating centers will be informed about the current status of recruitment and adverse events via a newsletter every month. Additionally, there will be regular contact between the principle investigator and the local investigators from the participating centers.

### Statistical analyses

The study results will be evaluated based on intention-to-treat analysis. Data concerning the 1, 2, and 5 years follow-up will be analyzed for both study groups on an intention-to-treat manner by student t-test (normal distribution) or Mann Whitney U-test (skewed distribution). To test for normality, the Kolmogorov-Smirnov test will be used. The Chi-square test will be used for binomial data. All probability values are two-tailed. *P* <0.05 will be considered significant. Obliteration rates will be presented as Kaplan Meier curves, including censoring in case of loss to follow-up.

An interim analysis will be performed after treatment and at 4 weeks follow-up for the first 80 patients to monitor the progression of the study. Final analysis will be performed after follow-up of the last patient included in this study is completed.

### Publication of data

Data will be published after all patients had a follow-up period of 1 year, regardless of the outcome of the study. Separate publication of data on pain, initial technical success and short-term follow-up can be published earlier. Long-term results will be published after 2 or 5 years follow-up. Co-authorship will be assigned according to the ‘Recommendations for the Conduct, Reporting, Editing, and Publication of Scholarly Work in Medical Journals’ of the International Committee of Medical Journal Editors [23].

### Definitions

Anatomical success is defined as occlusion of the treated SSV segment, measured with duplex ultrasonography.

Clinical success is defined as the objective improvement of clinical outcome after treatment, measured with the Venous Clinical Severity Score (VCSS).

Initial technical success is defined as the safe placement of the device at the predefined distance from the SPJ, treatment of the SSV without technical problems and occlusion of the treated SSV directly after treatment.

#### Failure of treatment

Failure of treatment is defined as follows:

1. Type 1 (non-occlusion): the treated vein failed to occlude initially and never occluded during the follow-up.
2. Type 2 (recanalisation): the treated vein occluded directly after treatment, but recanalized, partially (>10 cm) or completely, at a later time point during follow-up.
  - Type 2a: recanalization of the entire treated segment of the vein.
  - Type 2b: partial recanalization (open segment >10 cm) [24].

Post-procedural complications (complications occurring within 30 days after treatment) are defined as follow:

1. Major complication: deep venous thrombosis, pulmonary embolism, skin burn, or nerve injury.
2. Minor complication: ecchymosis, superficial phlebitis, hyperpigmentation, induration, wound infection of the puncture site, or prolonged pain >1 week.
3. Sclerosans-related complications.

The duration of procedure includes the time of the procedure, starting from puncture of the vein to the extracting of the catheter, skin-to-skin contact.

## Discussion

The introduction of minimal invasive endovenous ablation techniques has revolutionized the treatment of saphenous vein insufficiency. Different endothermal techniques have been introduced and tested in many prospective and randomized studies. Although most research is performed in GSVs, limited data on SSV is available. The results of endothermal techniques have proven to be excellent with a long-term anatomical success rate over 90%; therefore, the aim for future studies, especially in SSV, is diminishing heat-related complications, for example, pain and nerve injury, without compromising anatomical and clinical success. Until now the gold standard for the treatment of SSV insufficiency remains unclear.

This trial is designed to compare the anatomical success at 1 year after MOCA™ with RFA.

A secondary aim is to investigate whether MOCA™ is associated with a significant reduction in post-procedural pain. A major point of discussion is the choice for RFA above EVLA techniques. Although the meta-analysis of Van de Bos *et al*. showed the superiority of endothermal ablation (EVLA) in terms of anatomical success at 1 to 5 years follow-up [10], the more recent RCT of Rasmussen *et al*. has proven that the results of RFA using the ClosureFast device are at least similar to EVLT. In this randomized trial, RFA was associated with significantly less post-procedural pain compared to EVLA [24]. Furthermore, an important consideration in choosing ClosureFast is the uniformity of this technique, while in the case of ELVA, discussion about different tip designs and wavelengths might occur.

It has been proven that minimally invasive techniques will lead to a lower incidence of nerve injury in the SSV compared to conventional surgery (11% versus 28%). However, sural nerve injury is still considered a major and potentially underreported complication [25, 26]. Due to the fact that no heat and tumescent is used in MOCA™, nerve injury after SSV ablation might become a redundant complication.

In conclusion, the MESSI trial is a multicenter randomized controlled trial that aims to show a similar anatomical success of MOCA compared to RFA. Additionally, we hypothesize that this is accompanied by comparable clinical success and a reduction in post-procedural pain after MOCA™ compared with RFA.

### Trial status

The MESSI trial began including participants in the second quarter of 2014. We expect that inclusion will be completed in the third quarter of 2015.

## Abbreviations

AE: adverse event
AVVQ: Aberdeen Varicose Vein Questionnaire
CEAP: clinical etiology anatomy pathophysiology
CRF: case report form
DSMB: Data Safety Monitoring Board
eGFR: estimated glomerular filtration rate
EVLA: endovenous laser ablation
GSV: great saphenous vein
IQR: interquartile range
MOCA™: mechanochemical endovenous ablation
RFA: radiofrequency ablation
SF-36: Short Form-36
SPJ: saphenopoplitial junction
SSV: small saphenous vein
VAS: visual analog scale
VCSS: Venous Clinical Severity Score.

## References

[1] Andreozzi GM, Cordova RM, Scomparin A, Martini R, D’Eri A, Andreozzi F (2005). Quality of life working group on vascular medicine of SIAPAV. Quality of life in chronic venous insufficiency. An Italian pilot study of the Triveneto Region. Int Angiol.

[2] Callam MJ (1994). Epidemiology of varicose veins. Br J Surg.

[3] Almgren B, Eriksson E (1990). Valvular incompetence in superficial, deep and perforator veins of limbs with varicose veins. Acta Chir Scand.

[4] Engelhorn CA, Engelhorn AL, Cassou MF, Salles-Cunha SX (2005). Patterns of saphenous reflux in women with primary varicose veins. J Vasc Surg.

[5] Samuel N, Carradice D, Wallace T, Mekako A, Hatfield J, Chetter I (2013). Randomized clinical trial of endovenous laser ablation versus conventional surgery for small saphenous varicose veins. Ann Surg.

[6] Ikponmwosa A, Bhasin N, Weston MJ, Berridge DC, Scott DJ (2010). Outcome following saphenopopliteal surgery: a prospective observational study. Phlebology.

[7] O’Hare JL, Vandenbroeck CP, Whitman B, Campbell B, Heather BP, Earnshaw JJ, Joint Vascular Research Group (2008). A prospective evaluation of the outcome after small saphenous varicose vein surgery with one-year follow-up. J Vasc Surg.

[8] Allegra C, Antignani PL, Carlizza A (2007). Recurrent varicose veins following surgical treatment: our experience with five years follow-up. Eur J Vasc Endovasc Surg.

[9] Trip-Hoving M, Verheul J, Van Sterkenburg SMM, De Vries WR, Reijnen MMPJ (2009). Endovenous laser ablation of the small saphenous vein; short-term results and patient satisfaction. Photomed Laser Surg.

[10] Van den Bos R, Arends L, Kockaert M, Neumann M, Nijsten T (2009). Endovenous therapy of lower extremity varicosities: a meta-analysis. J Vasc Surg.

[11] Shepherd AC, Gohel MS, Brown LC, Metcalfe MJ, Hamish M, Davies AH (2010). Randomized clinic trial of VNUS ClosureFAST radiofrequency ablation versus laser for varicose veins. Br J Surg.

[12] Proebstle TM, Gul D, Kargl A, Knop J (2003). Endovenous laser treatment of the lesser saphenous vein with a 940-nm diode laser: early results. Dermatol Surg.

[13] Creton D, Pichot O, Sessa C, Proebstle TM (2010). Radiofrequency-powered segmental thermal obliteration carried out with the ClosureFast procedure: results at 1 year. Ann Vasc Surg.

[14] Boersma D, Van Eekeren RRJP, Werson DAB, De Vries JPPM, Reijnen MMJP (2013). Mechanochemical endovenous ablation of small saphenous vein insufficiency using the ClariVein® device: one-year results of a prospective series. Eur J Vasc Endovasc Surg.

[15] Elias S, Raines JK (2012). Mechanochemical tumescentless endovenous ablation: final results of the initial clinical trial. Phlebology.

[16] Van Eekeren RRJP, Boersma D, Elias S, Holewijn S, Werson DAB, De Vries JPPM, Reijnen MMJP (2011). Mechanochemical endovenous ablation of great saphenous vein incompetence using the ClariVein® device: a safety study. J Endovasc Ther.

[17] Bishawi M, Bernstein R, Boter M, Draugh D, Gould C, Hamilton C, Koziarski J (2013). Mechanochemical ablation in patients with chronic venous disease: a prospective multicenter report. Phlebology.

[18] Kistner RL, Eklof B, Masuda EM (1996). Diagnosis of chronic venous disease of the lower extremities: the “CEAP” classification. Mayo Clin Proc.

[19] Rutherford RB, Padberg FT, Comerota AJ, Kistner RL, Meissner MH, Moneta GL (2000). Venous severity scoring: an adjunct to venous outcome assessment. J Vasc Surg.

[20] Labropoulos N, Tiongson J, Pryor L, Tassiopoulos AK, Kang SS, Mansour MA, Baker WH (2003). Definition of venous reflux in lower extremity veins. J Vasc Surg.

[21] Proebstle TM, Vago B, Alm J, Gockeritz O, Lebard C, Pichot O (2008). Treatment of the incompetent great saphenous vein by endovenous radiofrequency powered segmental thermal ablation: first clinical experience. J Vasc Surg.

[22] Klem TM, Sybrandy JE, Wittens CH, Essink Bot ML (2009). Reliability and validity of the Dutch translated Aberdeen varicose vein questionnaire. Eur J Vasc Endovasc Surg.

[23] International Committee of Medical Journal Editors (2013). Recommendations for the Conduct, Reporting, Editing, and Publication of Scholarly Work in Medical Journals.

[24] Rasmussen LH, Lawaetz M, Bjoern L, Vennits B, Blemings A, Eklof B (2011). Randomized clinical trial of endovenous laser ablation, radiofrequency ablation, foam sclerotherapy and surgical stripping for great saphenous varicose veins. Br J Surg.

[25] O’Hare JL, Vandenbroeck CP, Whitman B, Campbell B, Heather BP, Earnshaw JJ (2008). A prospective evaluation of the outcome after small saphenous varicose vein surgery wtih one-year follow-up. J Vasc Surg.

[26] Huisman LC, Bruins RMG, Van den Berg M, Hissink RJ (2009). Endovenous laser ablation of the small saphenous vein: prospective analysis of 150 patients, a cohort study. Eur J Vasc Endovasc Surg.

